# Double-Bundle Versus Single-Bundle Anterior Cruciate Ligament (ACL) Reconstruction: A Systematic Review and Meta-Analysis of Knee Stability Outcomes

**DOI:** 10.7759/cureus.75352

**Published:** 2024-12-09

**Authors:** Abdelfatah M Elsenosy, Ahmed Elnewishy, Karim Rezk, Radwa A Delewar, Hagar Teama, Aya M Abdelfatah

**Affiliations:** 1 Trauma and Orthopaedics, University Hospitals Dorset National Health Service (NHS) Foundation Trust, Poole, GBR; 2 Trauma and Orthopaedics, Royal Berkshire Hospital, Reading, GBR; 3 Trauma and Orthopaedics, Airedale National Health Service (NHS) Foundation Trust, West Yorkshire, GBR; 4 Pharmacy, Alexandria University, Alexandria, EGY; 5 Pharmacy, Kafr El Sheikh General Hospital, Kafr El Sheikh, EGY; 6 Dentistry, Tanta University, Tanta, EGY

**Keywords:** acl reconstruction, double-bundle, ikdc, knee stability, lachman test, lysholm score, meta-analysis, pivot-shift test, single-bundle

## Abstract

This systematic review and meta-analysis compares the effectiveness of single-bundle (SB) and double-bundle (DB) ACL reconstruction techniques in improving knee stability and functional outcomes in patients with ACL injuries. A structured search across PubMed, Scopus, Google Scholar, and the Cochrane Library identified studies comparing SB and DB ACL reconstructions. Ten studies met the inclusion criteria, including randomized controlled trials, prospective, and retrospective studies. The primary outcomes analyzed were the International Knee Documentation Committee (IKDC) subjective score, Lysholm score, Lachman test, and pivot-shift test results. Meta-analytic methods included calculating standardized mean differences (SMDs) and odds ratios (ORs) with 95% confidence intervals (CIs) alongside assessments of heterogeneity using the I² statistic.

The meta-analysis showed no significant difference between SB and DB techniques for IKDC subjective scores (SMD: -0.14, 95% CI: -0.68 to 0.39, p = 0.59) or Lysholm scores (SMD: -0.18, 95% CI: -0.39 to 0.02, p = 0.08). Lachman test results also indicated no significant differences between techniques (pooled OR: 1.02, 95% CI: 0.70-1.47, p = 0.92). Pivot-shift test outcomes similarly revealed comparable rotational stability (OR: 1.00, 95% CI: 0.70-1.43, p = 1.00). Moderate heterogeneity was observed across analyses (I² = 37%-43%), reflecting variations in study designs and patient populations.

SB and DB ACL reconstruction techniques achieve similar functional outcomes and knee stability, with no significant differences in Lachman test results, pivot-shift outcomes, or patient-reported measures. Further research with standardized methodologies is needed to verify these findings across diverse populations.

## Introduction and background

The anterior cruciate ligament (ACL) is an essential structure within the knee that provides balance during physical activities, specifically in sports requiring pivoting, jumping, or rapid directional changes. ACL injuries are some of the most common ligament injuries, often caused by noncontact mechanisms such as sudden stops or directional shifts. The incidence of ACL injuries has increased, specifically in active and athletic populations, necessitating advancements in diagnostic and treatment methods [1].

Surgical reconstruction remains the gold standard for treating ACL injuries, aiming to restore knee stability and function. Techniques such as the use of autografts (e.g., bone-patellar tendon-bone, BTB, or hamstring tendons) are common, offering biomechanical properties similar to the native ACL. Innovations such as suture tape augmentation and bridge-enhanced ACL repair have emerged to enhance results, improve graft stability, and potentially reduce donor-site morbidity [2,3]. Postoperative rehabilitation is crucial for successful outcomes, focusing on restoring range of motion, strength, and neuromuscular control. Preoperative rehabilitation, or prehabilitation, has demonstrated advantages in enhancing quadriceps strength and physical readiness before surgery, which can result in improved postoperative recovery [4].

Despite advancements, challenges remain, such as high rates of reinjury and the onset of osteoarthritis in the long term. Novel approaches like 3D bioprinting and tissue engineering are under exploration to enhance ligament repair and regeneration, offering promise for the future of ACL treatment [5].

ACL reconstruction plays a pivotal role in restoring knee stability, specifically in patients who desire to resume active lifestyles or sports involving pivoting and cutting movements. The primary goal of reconstruction is to replicate the biomechanical properties of the native ACL, minimizing knee instability and reducing the risk of secondary injuries such as meniscal tears or cartilage damage [6]. Graft selection, including autografts (BTB or hamstring tendon) or allografts, significantly influences the success of ACL reconstruction. Autografts remain the gold standard due to their superior integration and lower rejection rates, while more recent approaches like suture tape augmentation aim to improve graft stability during the early recovery stages [3]. Advanced surgical techniques, including the “all-inside” technique, offer reduced surgical trauma and better cosmetic results while ensuring secure fixation of the graft. These techniques allow for quicker functional recovery and reduced donor-site morbidity, making them increasingly popular in clinical practice [7].

Single-bundle (SB) ACL reconstruction remains one of the most widely practiced techniques for treating anterior cruciate ligament injuries. The surgical procedure generally involves using autografts, such as hamstring tendons or BTB grafts. These grafts are secured using both the transtibial or anteromedial (AM) portal techniques. The AM portal technique has shown improved rotational stability compared to the transtibial technique, emphasizing the importance of precise tunnel placement for optimal outcomes [8]. Clinical outcomes of SB reconstruction have been extensively studied, showing significant improvements in knee stability and function. Postoperative tests, including the Lachman and pivot-shift tests, typically show restored anterior stability, with high rates of patient satisfaction and return to preinjury activity levels. Functional scores such as the Lysholm score and International Knee Documentation Committee (IKDC) score have also highlighted the effectiveness of this technique in enhancing knee function and quality of life [9].

Despite its success, one issue of SB reconstruction is its potential difficulty in controlling rotational instability, which is often better addressed by double-bundle (DB) reconstruction. Studies have noted that while SB techniques provide adequate anterior stability, their ability to replicate the complex biomechanics of the ACL may fall short compared to DB techniques. However, when the femoral and tibial tunnels are precisely placed, the long-term outcomes of SB techniques can be comparable to those of DB techniques [10].

DB ACL reconstruction represents a significant advancement in knee surgery, designed to replicate the natural anatomy of the anterior cruciate ligament by reconstructing both the AM and posterolateral (PL) bundles. This approach aims to restore knee stability more effectively than SB reconstruction, specifically for rotational stability, which is important in active individuals and athletes. Research has shown that DB reconstruction can reduce knee laxity and improve control during pivoting movements, addressing the limitations of SB techniques in handling rotational instability [11].

Clinical studies have highlighted the durability and effectiveness of DB reconstruction in providing stability and reducing reinjury rates. For example, DB reconstructions are associated with lower graft failure rates, particularly in younger, active populations, making them a preferred choice in complex knee injuries [12]. However, randomized controlled trials have not always shown significant differences in long-term functional outcomes between DB and SB techniques. Both methods restore knee function effectively, suggesting that the benefits of DB reconstruction might be more context-dependent [13]. Research suggests that SB reconstruction achieves similar long-term clinical outcomes as DB reconstruction, with both techniques demonstrating improvements in patient-reported outcomes, knee laxity, and return-to-sport rates [14]. The comparison between DB and SB ACL reconstruction is critical because of their differing approaches to restoring knee stability and function.

## Review

Review objective

The objective of this review is to evaluate and compare the clinical outcomes, knee stability, and functional performance associated with DB and SB ACL reconstruction techniques.

Methods

Search Strategy

A comprehensive search was performed in November 2024 using PubMed, Scopus, Google Scholar, and the Cochrane Library to identify studies comparing DB and SB anterior cruciate ligament (ACL) reconstruction techniques in relation to knee stability. The search employed a combination of MeSH terms and keywords, including “double-bundle ACL reconstruction”, “single-bundle ACL reconstruction”, “knee stability”, and “pivot-shift test”. Boolean operators (AND, OR) were applied to optimize the results, with filters set to limit the search to English-language studies published in the last five years. Furthermore, the reference lists of selected articles were manually reviewed to identify any relevant studies not captured in the initial database search.

Eligibility Criteria

The inclusion and exclusion criteria for study selection are outlined in Table 1.

**Table 1:** Inclusion and exclusion criteria for studies comparing SB and DB ACL reconstruction techniques ACL: anterior cruciate ligament; DB: double bundle; IKDC: International Knee Documentation Committee; RCTs: randomized controlled trials; SB: single bundle

Criteria	Description
Inclusion criteria	RCTs, cohort studies, or observational studies comparing DB and SB ACL reconstruction strategies
	Reported at least one primary outcome (e.g., IKDC subjective score, Lysholm score, Lachman test, and pivot-shift test)
	Published in English
Exclusion criteria	Studies without a direct comparison between DB and SB ACL reconstruction techniques
	Case reports, editorials, opinion pieces, or conference abstracts
	Studies lacking sufficient outcome data or not available in English

Outcome Measures

The primary outcomes assessed in the review included the IKDC subjective score, which measures patient-reported knee function; the Lysholm score, which evaluates knee symptoms and functionality; the Lachman test, which assesses anterior knee stability; and the pivot-shift test, which evaluates rotational stability. The secondary outcomes included rates of graft failure, return-to-sport rates, adverse events such as infections or meniscal injuries, and radiographic evidence of osteoarthritis progression.

Data Extraction and Quality Assessment

Data were extracted independently by two reviewers using a standardized form to capture information on study design, patient demographics, interventions, outcomes, and follow-up periods. Any disagreements were resolved by consensus or by consulting a third reviewer. The quality of included studies was assessed using the Risk Of Bias In Non-randomized Studies (ROBINS-I) tool, which evaluates the risk of bias across multiple domains, including confounding, selection bias, and outcome reporting. Each study was classified as having low, moderate, serious, or critical risk of bias.

Statistical Analysis

Statistical analysis was conducted using RevMan Version 5.4 software (The Cochrane Collaboration, London, UK). For continuous outcomes, such as IKDC, Lysholm, Lachman, and pivot-shift test results, standardized mean differences (SMDs) with 95% confidence intervals (CIs) were calculated. Heterogeneity among studies was assessed using the I² statistic, with values above 50% indicating substantial heterogeneity. A fixed and random-effects model was applied to account for variability between the studies. Funnel plots and Egger’s test were used to evaluate publication bias, with statistical significance set at p < 0.05.

Results

Search and Study Selection

**Figure 1:**
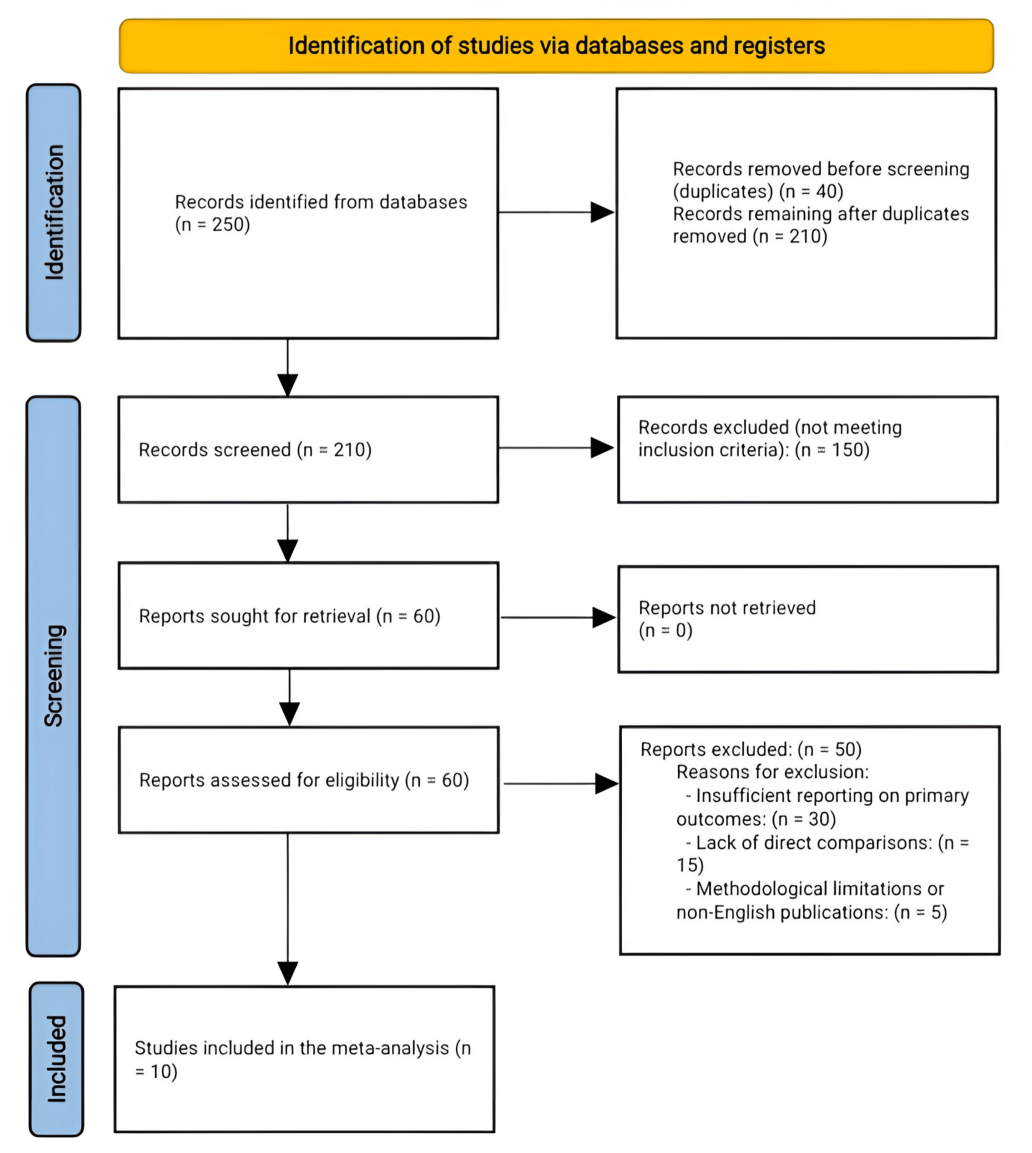
PRISMA flowchart showing the process of selecting studies, comparing SB and DB ACL reconstruction techniques ACL: anterior cruciate ligament; DB: double bundle; PRISMA: Preferred Reporting Items for Systematic Reviews and Meta-Analyses; SB: single bundle Image credit: This is an original image created by the author Abdelfatah M. Elsenosy

A structured search identified 250 records related to comparing DB and SB anterior cruciate ligament (ACL) reconstruction techniques for knee stability. After removing duplicates, 210 records remained for screening. During the title and abstract review, 150 studies were excluded for not meeting the specific inclusion criteria, which required direct comparisons between DB and SB techniques. Following this, 60 full-text articles were assessed for eligibility. Of these, 50 studies were excluded due to insufficient reporting on primary outcomes (e.g., IKDC subjective score, Lysholm score, Lachman test, and pivot-shift test), lack of direct comparisons, methodological limitations, or non-English publications. Ultimately, 10 studies met all inclusion criteria and were included in the final meta-analysis. Figure 1 provides a detailed Preferred Reporting Items for Systematic Reviews and Meta-Analyses flowchart of this study selection process, illustrating the stages of identification, screening, eligibility, and inclusion.

Study Characteristics

This meta-analysis included 10 studies encompassing randomized controlled trials, prospective observational studies, and retrospective comparative studies, all evaluating anterior cruciate ligament (ACL) reconstruction using DB or SB techniques. The sample sizes ranged from 36 to 330 participants, with a total of 1,125 patients analyzed (550 in the DB group and 575 in the SB group). The patient population is primarily comprised of active individuals aged 14-50 years, many of whom are engaged in sports such as football, basketball, and skiing, highlighting the high prevalence of ACL injuries in physically demanding activities. Most participants had primary ACL injuries, with no significant differences observed in the mechanisms of injury between the DB and SB groups.

The interventions compared included the DB technique, which anatomically reconstructs the AM and PL bundles through separate tunnels, and the SB technique, which employs a single graft placed in a central tunnel. The choice between these techniques often reflects clinical considerations such as the patient’s activity level, anatomical variations, and surgeon expertise. Follow-up durations ranged from 2 to 10 years, providing robust data on short- and long-term outcomes. Primary outcomes, including the IKDC subjective score and the Lysholm score, captured patient-reported knee function and symptom resolution, while objective assessments like the Lachman and pivot-shift tests evaluated anterior stability and rotational control, respectively.

Secondary outcomes, including graft failure rates, meniscal injury incidence, return-to-sport rates, and adverse events (e.g., infections, reruptures), were also examined, alongside radiographic evidence of osteoarthritis progression. Functional recovery was further assessed using performance tests, such as the single-leg hop test. The findings underscore the importance of tailoring surgical techniques to individual patient needs, guided by current evidence and clinical guidelines. By aligning interventions with patient-specific factors, clinicians can optimize outcomes, particularly in athletic populations where functional recovery and return to sport are critical priorities. Table 2 provides a detailed comparison of the outcomes and a comprehensive analysis of the data.

**Table 2:** Summary of study characteristics, including sample size, patient demographics, interventions, and primary outcomes for SB and DB ACL reconstruction techniques ACL: anterior cruciate ligament; ACLR: anterior cruciate ligament reconstruction; AM: anteromedial; A-SB: anatomical single bundle; DB: double bundle; DCE-MRI: Dynamic contrast-enhanced magnetic resonance imaging; F-DB: functional double bundle; HT: hamstring tendon; IKDC-SKF: International Knee Documentation Committee subjective knee form; KOOS: Knee injury and Osteoarthritis Outcome Score; LET: lateral extra-articular tenodesis; OA: osteoarthritis; PL: posterolateral; PT: patellar tendon; QoL: quality of life; RT: remnant-tensioning single bundle; SB: single bundle

Category	Irrgang et al. [13]	Balasingam et al. [14]	Kim et al. [15]	Legnani et al. [16]	Toker et al. [17]	Aga et al. [18]	Reddy et al. [19]	Zhang et al. [20]	Mohtadi and Chan [21]	Mayr et al. [22]
Study design	Randomized clinical trial comparing anatomic SB vs. DB ACL reconstruction	Randomized clinical trial comparing anatomic SB vs. DB ACL reconstruction	Prospective, randomized controlled trial comparing remnant-tensioning SB (RT-SB) vs. DB ACL reconstruction	Retrospective comparative study comparing SB ACL reconstruction with LET (SB+LET) vs. DB ACL reconstruction	Retrospective comparative study evaluating SB vs. DB ACL reconstruction in adolescent elite athletes	Prospective randomized controlled trial comparing anatomic SB vs. DB ACL reconstruction	Prospective randomized double-blind study comparing SB vs. DB ACL reconstruction	Prospective randomized controlled trial comparing functional DB (F-DB) vs. anatomical SB (A-SB) ACL reconstruction	Randomized clinical trial comparing PT, HT, and DB ACL reconstructions	Prospective randomized controlled trial comparing SB vs. DB ACL reconstruction
Sample size	57 participants (29 DB, 28 SB), aged 14–50 years	105 patients (53 DB, 52 SB). 10-year follow-up: 70 patients (39 DB, 31 SB)	67 patients randomized (RT-SB: 33; DB: 34); 54 completed follow-up	36 patients: SB+LET (n = 16), DB (n = 20)	89 patients (SB: 51, DB: 38)	116 patients (SB: 62, DB: 54)	65 patients enrolled; 60 completed the 5-year follow-up (SB: 30, DB: 30)	156 patients (78 F-DB, 78 A-SB)	330 patients; 315 (95%) completed 5-year follow-up	64 patients (SB: 30, DB: 34); 53 completed 5-year follow-up
Level of evidence	Level II	Level I	Level II	Level III	Level III	Level I	Level I	Level II	Level I	Level I
Patient demographics	Active individuals aged 14-50 years; DB: 23.1 ± 9.2 years, SB: 20.3 ± 4.3 years; 64% male	Median age: DB = 33 years (18-50), SB = 26 years (18-52). 67% male overall.	Mean age: RT-SB: 33.6 ± 9.5 years; DB: 29.1 ± 7.9 years. 80% male	Mean age: SB+LET: 26.8 ± 8.7 years, DB: 28.3 ± 9.2 years; 64% male	Age: SB: 15.4 ± 1.03 years, DB: 15.7 ± 1.3 years; football was the primary sport (70%)	Age: 18-40 years; SB: 66% male, DB: 87% male	Age: 17-40 years; predominantly male (SB: 28/2, DB: 30/0).	Age: 18+ years; 183 males, 147 females. Acute and chronic ACL injuries	Age: mean ~33 years; 183 males, 147 females	Age: mean: 38.5 ± 9.8 years; SB: 12 males, 13 females, DB: 15 males, 13 females
Intervention details	DB ACLR: graft split into 2 for AM and PL bundles; SB ACLR: single graft placed in a central tunnel	DB ACLR: separate tunnels for AM and PL bundles; SB ACLR: single tunnel centered in ACL footprint	RT-SB: preserved ACL remnant tensioned with SB graft; DB: separate tunnels for AM and PL bundles	SB+LET: autologous hamstring graft with lateral tenodesis; DB: AM and PL hamstring grafts	SB: single femoral and tibial tunnel with semitendinosus and gracilis grafts; DB: separate AM and PL bundles	SB: ipsilateral semitendinosus graft; DB: ipsilateral and contralateral grafts, separate tunnels	SB: anatomical placement of single femoral and tibial tunnels; DB: separate AM and PL bundles	F-DB: hamstring graft with preserved tibial insertion; AM and PL bundles fixed at different flexion angles; A-SB: single femoral and tibial tunnels	PT: central third of patellar tendon autograft; HT: hamstring autograft; DB: two-bundle hamstring autograft	SB: anatomical single femoral and tibial tunnels; DB: separate AM and PL bundles with individual tunnels
Follow-up duration	24 months	Median: 120 months (range: 112-134)	Mean: 28.7 ± 6.4 months	Mean: 6.2 years (range: 2-9 years)	Mean: SB: 53.1 ± 8.6 months, DB: 46.4 ± 9.1 months	2 years	5 years	2 years	5 years	5 years (mean: 63.2 ± 4.7 months)
Outcome measures	IKDC-SKF, KOOS, pivot-shift test, KT-1000, return-to-sport rates	IKDC-SKF, KOOS, pivot-shift test, KT-1000, return-to-sport rates	IKDC-SKF, KOOS, pivot-shift test, KT-1000, return-to-sport rates	IKDC-SKF, KOOS, pivot-shift test, KT-1000, return-to-sport rates	IKDC-SKF, KOOS, pivot-shift test, KT-1000, return-to-sport rates	IKDC-SKF, KOOS, pivot-shift test, KT-1000, return-to-sport rates	IKDC-SKF, KOOS, pivot-shift test, KT-1000, return-to-sport rates	IKDC-SKF, KOOS, pivot-shift test, KT-1000, return-to-sport rates	IKDC-SKF, KOOS, pivot-shift test, KT-1000, return-to-sport rates	IKDC-SKF, KOOS, pivot-shift test, KT-1000, return-to-sport rates
Results	Both groups achieved ~90 in IKDC-SKF; minor differences in laxity, graft retears, and meniscus injuries	DB showed no significant differences in functional tests but had minor radiographic OA progression	RT-SB showed improved vascularity but no significant difference in clinical outcomes compared to DB	No significant differences in functional or radiographic outcomes	SB had fewer complications and similar outcomes as DB	KOOS QoL was slightly higher in DB; graft failure rates comparable	No significant differences in IKDC or functional tests between SB and DB	F-DB showed slightly better functional scores and lower pivot-shift rates than A-SB	No significant differences in subjective or objective IKDC scores across groups	No significant differences in functional or radiographic outcomes
Subgroup analysis	No subgroup-specific findings of significance	No significant findings in subgroup analyses	No significant difference in DCE-MRI findings between autograft and allograft	No subgroup-specific results provided	Graft size (<7.5 mm vs. ≥7.5 mm): no effect on rerupture rates. Additional meniscus procedures: no significant differences in outcomes	- Blinded Patients: No significant differences in KOOS QoL scores. Patients without graft ruptures: similar outcomes between SB and DB groups	No significant subgroup-specific findings reported	Rotational stability: improved in F-DB group as shown by pivot-shift test. Graft diameter: no subgroup differences noted	Patients without reinjuries: ACL-QoL scores slightly higher in all groups but no significant differences	No subgroup-specific differences noted
Adverse events	Patellar fractures: 5 cases (8.8%) overall (DB: 2, SB: 3). Meniscus tears: DB: 1 (3.5%), SB: 4 (14.3%). Other: contralateral ACL tears (4 cases, ~7.1%)	Second-look surgery: DB: 4, SB: 6 (p = 0.28). Graft ruptures: DB: 1 case, SB: 0 cases. Meniscal injuries: 22% (both groups combined)	Cyclops lesions: RT-SB: 1 case; DB: 3 cases. Graft rupture: RT-SB: 2 cases; DB: 3 cases. No infections reported	No graft failures or major complications reported, and no postoperative stiffness or donor-site morbidity observed	Reruptures: total 21 cases (SB: 9, DB: 12). Contralateral injuries: SB: 4 cases, DB: 7 cases (p = 0.13). Deep joint infections: SB: 0, DB: 2 (5.2%).	Graft ruptures: SB: 8 (13%), DB: 3 (6%). Infections: 2 cases in each group. Meniscal injuries: 1 (SB) vs. 3 (DB). Reoperations: SB: 11 (17.7%), DB: 5 (9.3%)	SB group: anterior knee pain: 2 cases, terminal extension pain: 1 case. DB group: early infection: 1 case, sensory deficit: 1 case, terminal extension pain: 1 case	No graft ruptures in either group, minimal infections (one patient in F-DB), and no reoperations required	Kneeling pain (five years): PT: 10%, HT: 4%, DB: 2% (p = 0.029). Atraumatic graft failures: PT: 7%, HT: 9%, DB: 14% (p = 0.145)	Complications: No graft failures in either group, cyclops syndrome: SB: 2 cases, DB: 1 case, contralateral ACL rupture: SB: two cases, DB: 1 case
Conclusions	No significant differences in clinical outcomes between SB and DB ACLR at 24 months. Both achieved comparable stability and return to sports	DB technique was not superior to SB in long-term outcomes. No clinically significant differences in knee function, stability, or radiographic OA	RT-SB provided comparable stability and clinical outcomes to DB but demonstrated significantly better graft vascularity on DCE-MRI at one year postoperatively	Both SB+LET and DB techniques were effective for restoring rotational stability and functional outcomes. No significant differences in clinical outcomes or laxity between the two groups	No significant differences in clinical outcomes, reruptures, or complications between SB and DB techniques in adolescent elite athletes	No significant differences in KOOS QoL, clinical, or functional outcomes between SB and DB ACL reconstructions at two-year follow-up. Both techniques provided improved outcomes	No statistically significant differences between SB and DB techniques in terms of clinical outcomes or complications at five-year follow-up. Both techniques showed improvement compared to baseline	F-DB ACL reconstruction provided better clinical outcomes, including rotational stability, IKDC scores, and KOOS subscores, compared to A-SB. Both techniques significantly improved outcomes compared to baseline	No significant differences in ACL-QOL or clinical outcomes at five years. PT showed fewer reinjuries and graft failures but higher kneeling pain	No significant differences in clinical or functional outcomes between SB and DB techniques at five years. Both techniques showed similar improvement in subjective and objective measures

Quality Assessment of Included Studies

The quality of the included studies was assessed using the ROBINS-I tool, which evaluates the risk of bias across key domains such as confounding, participant selection, and outcome measurement. Each study was categorized as having low, moderate, or serious risk of bias, as detailed in Table 3.

**Table 3:** Risk of bias assessment for included studies using the ROBINS-I tool, which evaluates bias across key domains such as confounding, selection, and outcome measurement ROBINS-I: Risk Of Bias In Non-randomized Studies

Study	Bias due to confounding	Bias in selection of participants	Bias in classification of intervention	Bias due to deviations from intended interventions	Bias due to missing data	Bias in measurement of outcomes	Bias in selection of reported results	Overall risk of bias
Irrgang et al. [13]	Low	Low	Low	Low	Low	Low	Low	Low
Balasingam et al. [14]	Low	Low	Low	Low	Low	Low	Low	Low
Kim et al. [15]	Moderate	Low	Low	Low	Low	Moderate	Low	Moderate
Legnani et al. [16]	Moderate	Low	Low	Low	Low	Moderate	Low	Moderate
Toker et al. [17]	Moderate	Low	Low	Low	Low	Moderate	Low	Moderate
Aga et al. [18]	Low	Low	Low	Low	Low	Low	Low	Low
Reddy et al. [19]	Low	Low	Low	Low	Low	Low	Low	Low
Zhang et al. [20]	Low	Low	Low	Low	Low	Low	Low	Low
Mohtadi and Chan [21]	Low	Low	Low	Low	Low	Low	Low	Low
Mayr et al. [22]	Low	Low	Low	Low	Low	Low	Low	Low

Results of Meta-Analysis

**Figure 2:**
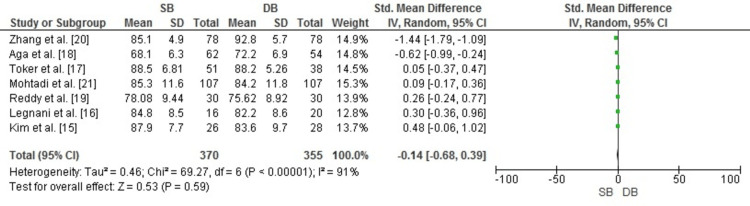
Forest plot showing the IKDC subjective scores comparing SB and DB ACL reconstruction techniques. Results include the SMD with 95% CI ACL: anterior cruciate ligament; CI: confidence interval; DB: double bundle; IKDC: International Knee Documentation Committee; IV: inverse variance; SB: single bundle; SD: standard deviation; SMD: standardized mean difference Image credit: This is an original image created by the author Abdelfatah M. Elsenosy

IKDC subjective score: To evaluate patient-reported knee function following anterior cruciate ligament (ACL) reconstruction, the IKDC subjective scores were analyzed. The meta-analysis results, presented in Figure 2, show no significant difference between DB and SB ACL reconstruction techniques (SMD: -0.14, 95% CI: -0.68 to 0.39, p = 0.59). However, substantial heterogeneity (I² = 91%) was observed, likely due to variations in study design, population characteristics, surgical techniques, and follow-up durations.

**Figure 3:**
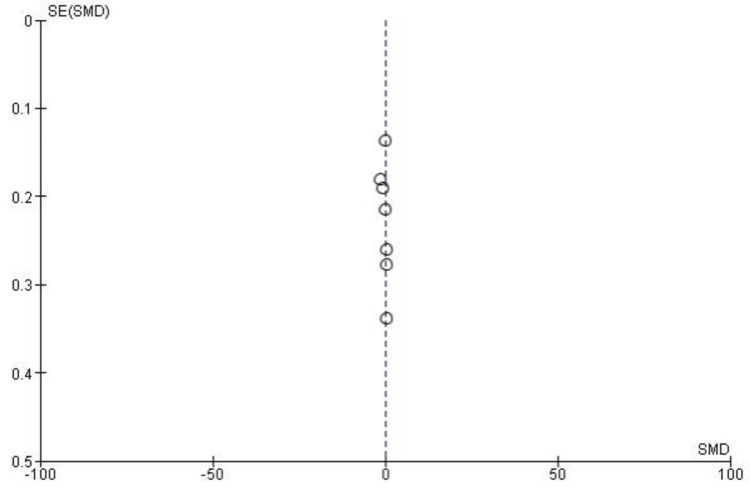
Funnel plot assessing publication bias for the IKDC subjective scores in studies comparing SB and DB ACL reconstruction techniques. The plot appears symmetrical around the SMD ACL: anterior cruciate ligament; DB: double bound; IKDC: International Knee Documentation Committee; SB: single bound; SE: standard error; SMD: standardized mean difference Image credit: This is an original image created by the author Abdelfatah M. Elsenosy

Publication bias for IKDC subjective score: To assess the potential for publication bias in the included studies, a funnel plot analysis was conducted for the IKDC subjective scores. As shown in Figure 3, the plot appears symmetrical around the SMD, indicating no significant publication bias. This finding was further supported by Egger’s test, which showed no statistical significance (p > 0.05).

**Figure 4:**
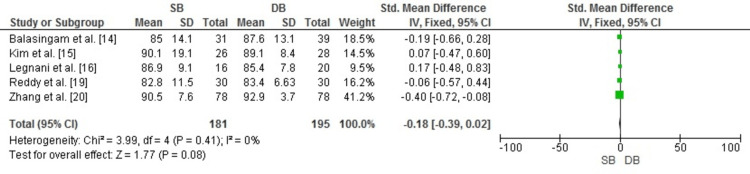
Forest plot comparing Lysholm knee scores for SB and DB ACL reconstruction techniques. Results are presented as SMD with 95% CI ACL: anterior cruciate ligament; CI: confidence interval; DB: double bundle; IV: inverse variance; SB: single bundle; SD: standard deviation; SMD: standard mean difference Image credit: This is an original image created by the author Abdelfatah M. Elsenosy

Lysholm score: The Lysholm score was used to evaluate knee symptoms and functionality following anterior cruciate ligament (ACL) reconstruction. As illustrated in Figure 4, the meta-analysis results indicate no statistically significant difference between DB and SB techniques (SMD: -0.18, 95% CI: -0.39 to 0.02, p = 0.08). Additionally, there was no evidence of heterogeneity (I² = 0%, p = 0.41), suggesting consistent findings across the included studies.

**Figure 5:**
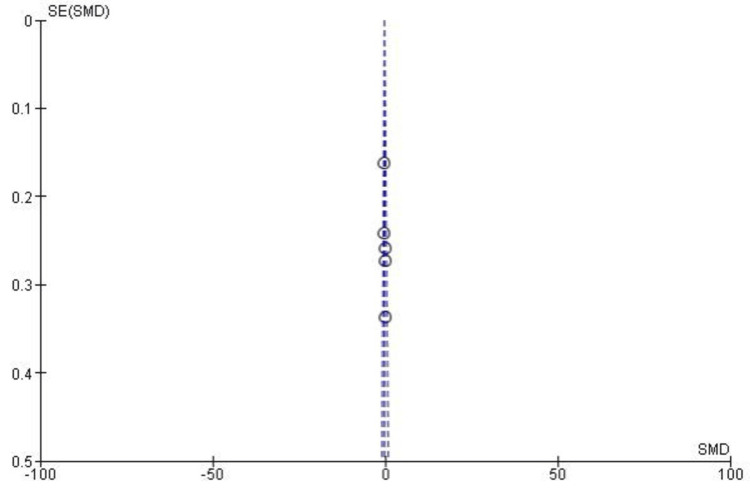
Funnel plot assessing publication bias for the Lysholm score in the meta-analysis of ACL reconstruction. The plot showed symmetrical distribution, indicating minimal bias. The SMD was used to evaluate effect size ACL: anterior cruciate ligament; SE: standard error; SMD: standardized mean difference Image credit: This is an original image created by the author Abdelfatah M. Elsenosy

Publication bias for Lysholm score: A funnel plot analysis was conducted to assess publication bias in the Lysholm score meta-analysis. As shown in Figure 5, the plot displayed a symmetrical distribution of studies around the central line, indicating minimal publication bias. This observation was further supported by Egger’s test, which yielded no statistically significant results (p > 0.05).

Lachman Test Results

**Figure 6:**
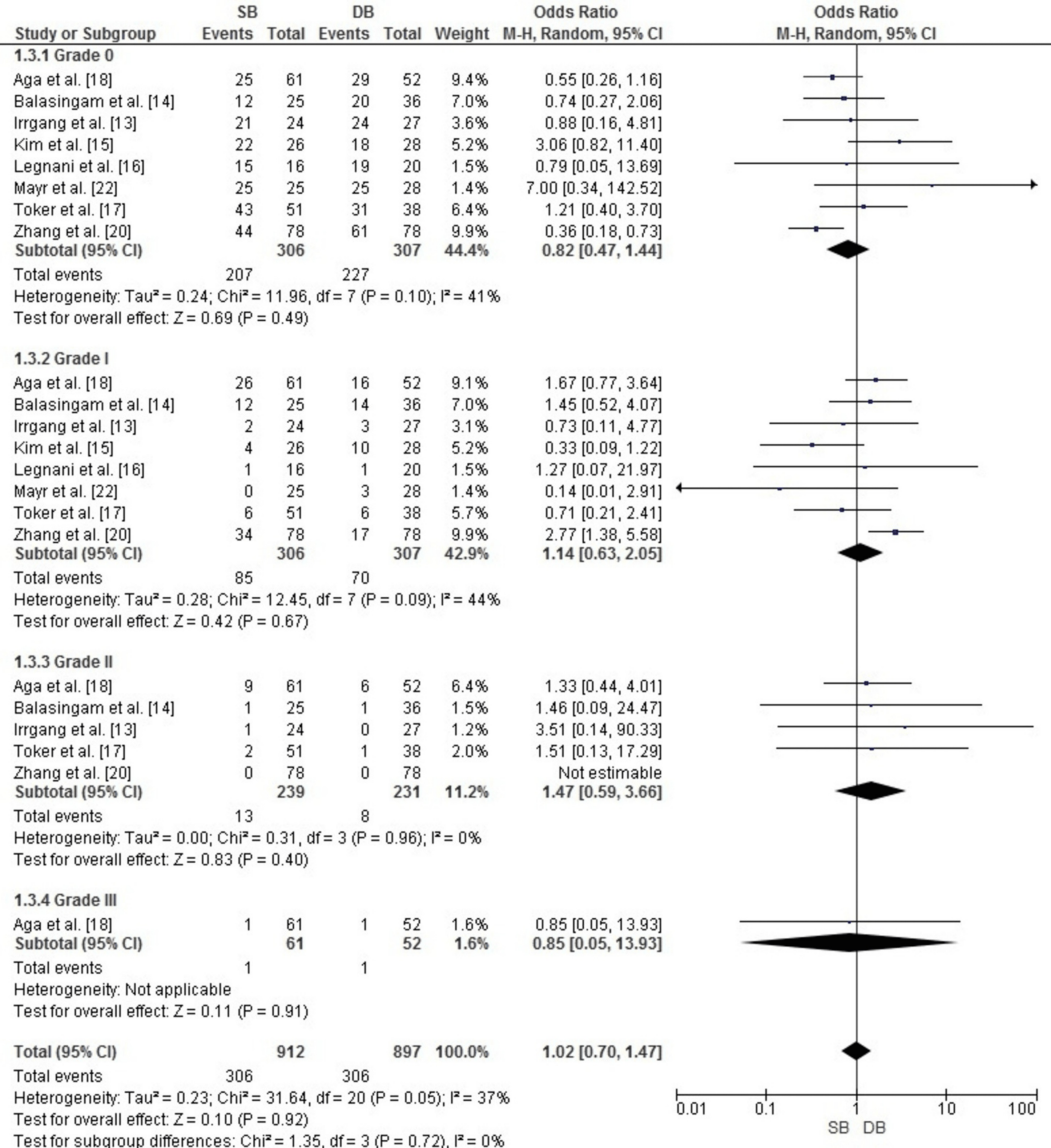
Forest plot showing the Lachman test results, which assess anterior knee stability, comparing SB and DB ACL reconstruction techniques. Results are reported as ORs with 95% CI ACL: anterior cruciate ligament; CI: confidence interval; DB: double bundle; M-H: Mantel-Haenszel; OR: odds ratio; SB: single bundle Image credit: This is an original image created by the author Abdelfatah M. Elsenosy

The Lachman test was used to assess anterior knee stability following anterior cruciate ligament (ACL) reconstruction. As shown in Figure 6, the meta-analysis found no significant overall difference in Lachman test outcomes between SB and DB ACL reconstruction techniques (pooled odds ratio, OR: 1.02, 95% CI: 0.70-1.47, p = 0.92). Subgroup analyses by grade also revealed no significant differences, including grade 0 (OR: 0.82, p = 0.49), grade I (OR: 1.14, p = 0.67), grade II (OR: 1.47, p = 0.40), and grade III (OR: 0.85, p = 0.91). Overall heterogeneity was low to moderate (I² = 37%), suggesting consistent findings across the studies included in the analysis.

Publication Bias for Lachman Test

**Figure 7:**
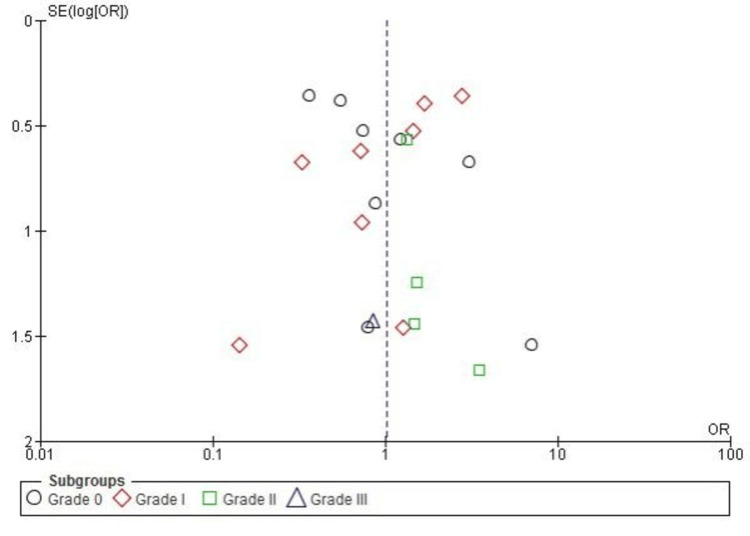
Funnel plot assessing publication bias for the Lachman test in ACL reconstruction. The plot shows minimal bias, with symmetrical distribution across grades 0-III. OR and SE of log(OR) were used ACL: anterior cruciate ligament; OR: odds ratio; SE: standard error Image credit: This is an original image created by the author Abdelfatah M. Elsenosy

Publication bias for the Lachman test meta-analysis was assessed using a funnel plot. As illustrated in Figure 7, the plot displayed a reasonably symmetrical distribution of studies across all grades (grades 0-III), indicating minimal publication bias. This observation was further supported by Egger’s test, which showed no statistically significant results (p > 0.05).

Pivot-Shift Test

**Figure 8:**
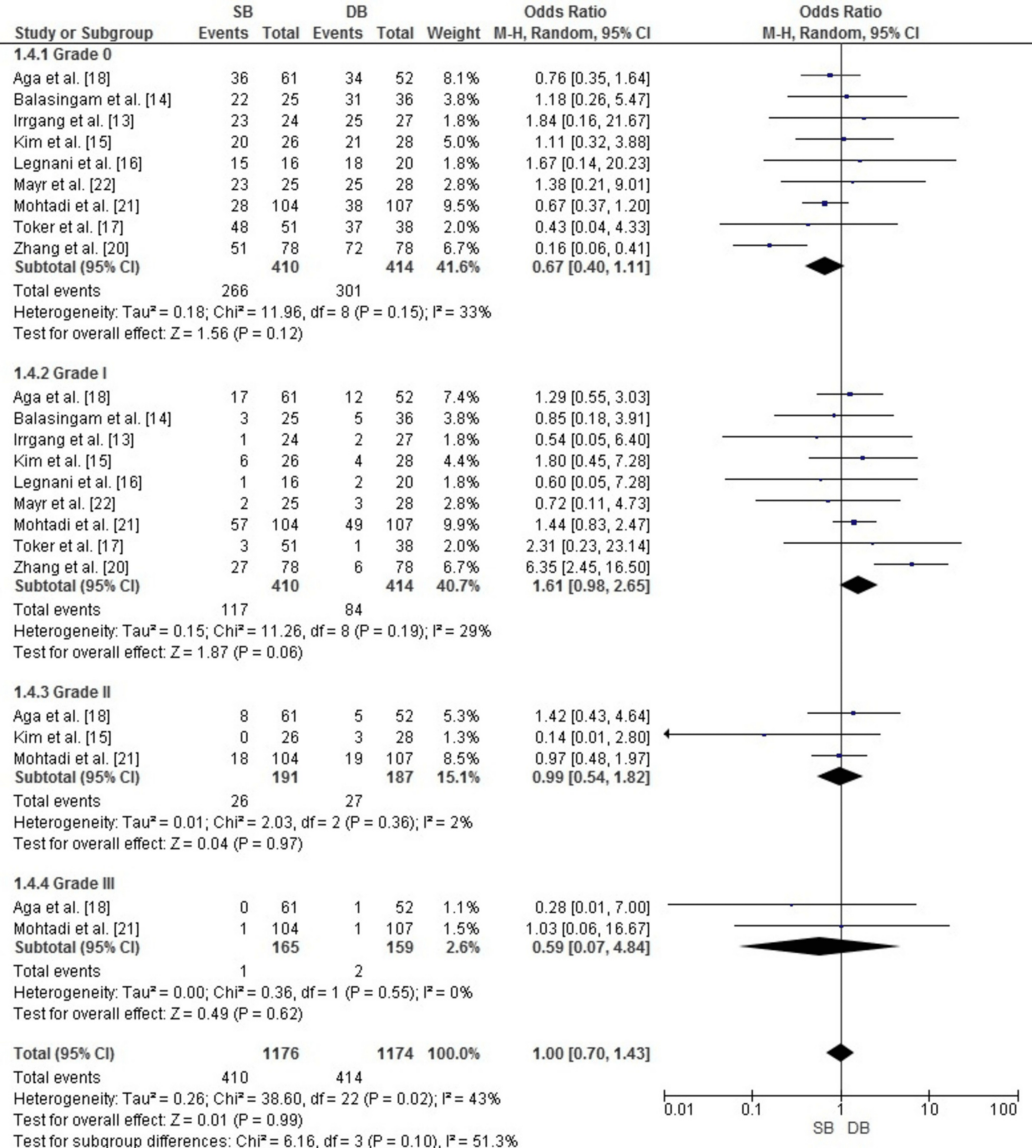
Forest plot showing pivot-shift test results, which assess rotational knee stability, comparing SB and DB ACL reconstruction techniques. Results are presented as ORs with 95% CI ACL: anterior cruciate ligament; CI: confidence interval; DB: double bundle; M-H: Mantel-Haenszel; OR: odds ratio; SB: single bundle Image credit: This is an original image created by the author Abdelfatah M. Elsenosy

The pivot-shift test was used to evaluate rotational knee stability following anterior cruciate ligament (ACL) reconstruction. As presented in Figure 8, the meta-analysis found no significant overall difference in pivot-shift test outcomes between SB and DB ACL reconstruction techniques (pooled OR: 1.00, 95% CI: 0.70-1.43, p = 1.00). Subgroup analyses revealed no significant differences for grade 0 (OR: 0.67, p = 0.12), grade I (OR: 1.61, p = 0.06), grade II (OR: 0.99, p = 0.97), or grade III (OR: 0.59, p = 0.62) outcomes. Overall heterogeneity was moderate (I² = 43%), indicating consistent findings across the included studies.

Publication Bias for Pivot-Shift Test

**Figure 9:**
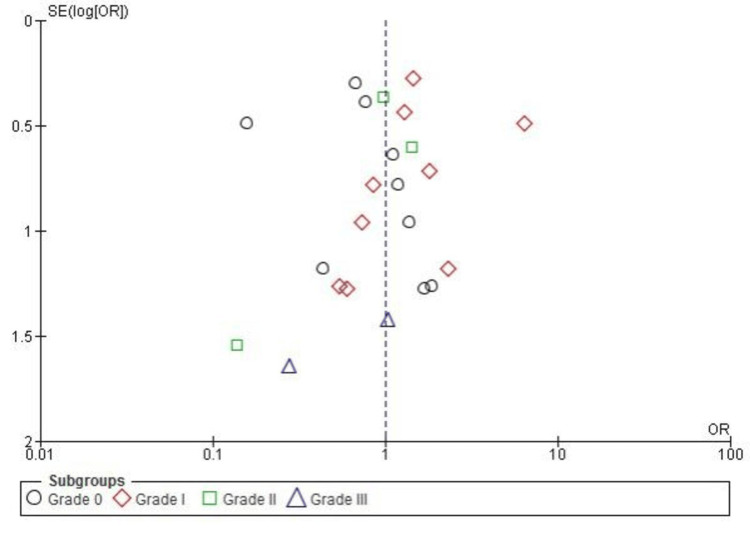
Funnel plot assessing publication bias for pivot-shift test results comparing SB and DB ACL reconstruction techniques. Results are presented as OR and SE of log(OR) ACL: anterior cruciate ligament; DB: double bundle; OR: odds ratio; SB: single bundle; SE: standard error Image credit: This is an original image created by the author Abdelfatah M. Elsenosy

Publication bias for the pivot-shift test meta-analysis was assessed using a funnel plot. As illustrated in Figure 9, the plot displayed a reasonably symmetrical distribution of studies across all subgroups (grades 0-III), indicating minimal publication bias. This observation was further supported by Egger’s test, which showed no statistically significant evidence of bias (p > 0.05).

Discussion

This systematic review and meta-analysis compared the outcomes of DB and SB anterior cruciate ligament (ACL) reconstruction strategies, focusing on knee stability, functional performance, and patient-reported outcomes. The findings indicate no statistically significant differences between the two techniques across key outcome measures, suggesting comparable clinical efficacy.

Several studies have found no significant benefits of DB techniques over SB strategies in terms of patient-reported outcomes or long-term functionality. For instance, Chen et al. [23] conducted a meta-analysis comparing mid- to long-term outcomes and concluded that DB reconstruction did not provide superior graft failure rates or osteoarthritis progression. Their findings emphasized that while DB techniques may offer better stability in controlled settings, the clinical outcomes are comparable to SB strategies.

Additionally, Järvelä et al. [24] performed a 10-year follow-up study and reported no significant differences in IKDC scores, Lysholm scores, or pivot-shift test results between DB and SB reconstructions. While DB techniques showed slightly fewer graft failures, this did not translate to improved patient satisfaction or functional recovery. In another prospective randomized study, Zhang et al. [25] found no significant differences in subjective or objective knee stability measures between the two strategies over a two-year follow-up period. Both techniques provided excellent outcomes, suggesting that the benefits of DB reconstruction may not be clinically significant in most cases.

Conversely, other research highlights the benefits of DB ACL reconstruction in enhancing knee stability and biomechanical performance compared to SB strategies. A systematic review of randomized controlled trials and meta-analyses suggests that DB techniques provide superior rotational stability as measured by pivot-shift tests and KT-1000 arthrometer readings. For example, a study by Mascarenhas et al. [26] demonstrated that DB reconstruction yielded significantly better control over rotational and anterior stability than SB reconstruction while maintaining comparable functional outcomes such as Lysholm and IKDC scores.

Similarly, Izawa et al. [27] reported reduced anteroposterior displacement in DB reconstruction patients compared to SB techniques. Their findings showed a mean difference of 1.2 mm for DB vs. 4.1 mm for SB in KT-2000 arthrometer readings, emphasizing the biomechanical superiority of DB in controlling instability. In a cadaveric biomechanical study, Ahn et al. [28] demonstrated that DB reconstruction with lateral extra-articular tenodesis restored knee stability more effectively across various angles of flexion compared to SB strategies. This finding underscores the enhanced control provided by DB techniques in complex knee injury situations.

The conflicting findings highlight that while DB reconstruction offers theoretical and biomechanical benefits, these do not always translate into better clinical outcomes. The decision to use DB vs. SB reconstruction should, therefore, consider factors such as patient activity levels, injury complexity, and surgeon expertise.

Limitations

Despite these findings, several limitations warrant consideration. Significant heterogeneity throughout studies, especially in IKDC results, highlights the variety of surgical techniques and patient populations. Furthermore, the studies reviewed typically involved active individuals, limiting the generalizability of the findings to nonathletic populations. Future research should focus on standardizing surgical techniques and exploring outcomes in diverse patient cohorts.

## Conclusions

This study provides robust evidence that DB and SB ACL reconstruction strategies achieve similar functional and stability outcomes. While DB reconstruction offers theoretical advantages in anatomical fidelity, these do not translate into superior clinical results. Both techniques remain viable options, with the choice largely dependent on surgeon expertise and individual patient factors. Further high-quality, long-term studies are needed to elucidate subtle differences in outcomes and inform surgical decision-making.

## References

[1] Mittal A, Jain S, Ambade R, Landge S (2022). Anterior cruciate ligament injury: a review. J Pharm Res Int.

[2] Murray MM, Fleming BC, Badger GJ (2020). Bridge-enhanced anterior cruciate ligament repair is not inferior to autograft anterior cruciate ligament reconstruction at 2 years: results of a prospective randomized clinical trial. Am J Sports Med.

[3] Benson DM, Hopper GP, Wilson WT, Mackay GM (2021). Anterior cruciate ligament reconstruction using bone-patellar tendon-bone autograft with suture tape augmentation. Arthrosc Tech.

[4] Carter HM, Littlewood C, Webster KE, Smith BE (2020). The effectiveness of preoperative rehabilitation programmes on postoperative outcomes following anterior cruciate ligament (ACL) reconstruction: a systematic review. BMC Musculoskelet Disord.

[5] Bakirci E, Guenat OT, Ahmad SS, Gantenbein B (2022). Tissue engineering approaches for the repair and regeneration of the anterior cruciate ligament: towards 3D bioprinted ACL-on-chip. Eur Cell Mater.

[6] Gerami MH, Haghi F, Pelarak F, Mousavibaygei SR (2022). Anterior cruciate ligament (ACL) injuries: a review on the newest reconstruction techniques. J Family Med Prim Care.

[7] Yang YT, Cai ZJ, He M, Liu D, Xie WQ, Li YS, Xiao WF (2022). All-inside anterior cruciate ligament reconstruction: a review of advance and trends. Front Biosci (Landmark Ed).

[8] Chen Y, Chua KH, Singh A (2015). Outcome of single-bundle hamstring anterior cruciate ligament reconstruction using the anteromedial versus the transtibial technique: a systematic review and meta-analysis. Arthroscopy.

[9] Moorthy V, Sayampanathan AA, Tan AH (2021). Superior postoperative stability and functional outcomes with anteromedial versus transtibial technique of single-bundle autologous hamstring anterior cruciate ligament reconstruction: a meta-analysis of prospective randomized controlled trials. Arthroscopy.

[10] Xu M, Gao S, Zeng C (2013). Outcomes of anterior cruciate ligament reconstruction using single-bundle versus double-bundle technique: meta-analysis of 19 randomized controlled trials. Arthroscopy.

[11] Oh JY, Kim KT, Park YJ (2020). Biomechanical comparison of single-bundle versus double-bundle anterior cruciate ligament reconstruction: a meta-analysis. Knee Surg Relat Res.

[12] Volpi P, Quaglia A, Carimati G, Galli M, Papalia R, Petrillo S (2019). Double bundle anterior cruciate ligament reconstruction: failure rate and patients-reported outcomes at 4-11 years of follow up. J Orthop.

[13] Irrgang JJ, Tashman S, Patterson CG (2021). Anatomic single vs. double-bundle ACL reconstruction: a randomized clinical trial-part 1: clinical outcomes. Knee Surg Sports Traumatol Arthrosc.

[14] Balasingam S, Karikis I, Rostgård-Christensen L, Desai N, Ahldén M, Sernert N, Kartus J (2022). Anatomic double-bundle anterior cruciate ligament reconstruction is not superior to anatomic single-bundle reconstruction at 10-year follow-up: a randomized clinical trial. Am J Sports Med.

[15] Kim JH, Oh E, Yoon YC, Lee DK, Lee SS, Wang JH (2021). Remnant-tensioning single-bundle anterior cruciate ligament reconstruction provides comparable stability to and better graft vascularity than double-bundle anterior cruciate ligament reconstruction in acute or subacute injury: a prospective randomized controlled study using dynamic contrast-enhanced magnetic resonance imaging. Arthroscopy.

[16] Ventura A, Legnani C, Terzaghi C, Borgo E (2012). Single- and double-bundle anterior cruciate ligament reconstruction in patients aged over 50 years. Arthroscopy.

[17] Toker B, Erden T, Dikmen G, Özden VE, Fıratlı G, Taşer Ö (2022). Clinical outcomes of single-bundle versus double-bundle ACL reconstruction in adolescent elite athletes: a retrospective comparative study. Acta Orthop Traumatol Turc.

[18] Aga C, Risberg MA, Fagerland MW, Johansen S, Trøan I, Heir S, Engebretsen L (2018). No difference in the KOOS quality of life subscore between anatomic double-bundle and anatomic single-bundle anterior cruciate ligament reconstruction of the knee: a prospective randomized controlled trial with 2 years' follow-up. Am J Sports Med.

[19] Reddy M, Babu M, Venugopal S, Deepak K, Sahu C, Sagar JVS (2019). A 5 year prospective double blind comparative study of ACL reconstruction using hamstring single bundle vs double bundle graft. Int J Orthop Sci.

[20] Zhang Q, Yang Y, Li J, Zhang H, Fu Y, Wang Y (2019). Functional double-bundle anterior cruciate ligament reconstruction using hamstring tendon autografts with preserved insertions is an effective treatment for tibiofemoral instability. Knee Surg Sports Traumatol Arthrosc.

[21] Mohtadi NG, Chan DS (2019). A randomized clinical trial comparing patellar tendon, hamstring tendon, and double-bundle ACL reconstructions: patient-reported and clinical outcomes at 5-year follow-up. J Bone Joint Surg Am.

[22] Mayr HO, Bruder S, Hube R, Bernstein A, Suedkamp NP, Stoehr A (2018). Single-bundle versus double-bundle anterior cruciate ligament reconstruction-5-year results. Arthroscopy.

[23] Chen H, Chen B, Tie K, Fu Z, Chen L (2018). Single-bundle versus double-bundle autologous anterior cruciate ligament reconstruction: a meta-analysis of randomized controlled trials at 5-year minimum follow-up. J Orthop Surg Res.

[24] Järvelä S, Kiekara T, Suomalainen P, Järvelä T (2017). Double-bundle versus single-bundle anterior cruciate ligament reconstruction: a prospective randomized study with 10-year results. Am J Sports Med.

[25] Zhang Z, Gu B, Zhu W, Zhu L (2014). Double-bundle versus single-bundle anterior cruciate ligament reconstructions: a prospective, randomized study with 2-year follow-up. Eur J Orthop Surg Traumatol.

[26] Mascarenhas R, Cvetanovich GL, Sayegh ET, Verma NN, Cole BJ, Bush-Joseph C, Bach BR Jr (2015). Does double-bundle anterior cruciate ligament reconstruction improve postoperative knee stability compared with single-bundle techniques? A systematic review of overlapping meta-analyses. Arthroscopy.

[27] Izawa T, Okazaki K, Tashiro Y (2011). Comparison of rotatory stability after anterior cruciate ligament reconstruction between single-bundle and double-bundle techniques. Am J Sports Med.

[28] Ahn JH, Koh IJ, McGarry MH, Patel NA, Lin CC, Lee TQ (2021). Double-bundle anterior cruciate ligament reconstruction with lateral extra-articular tenodesis is effective in restoring knee stability in a chronic, complex anterior cruciate ligament-injured knee model: a cadaveric biomechanical study. Arthroscopy.

